# The New York Homœopathic Union

**Published:** 1897-01

**Authors:** Charles H. Young

**Affiliations:** Secretary, pro tem.


### THE NEW YORK HOMŒOPATHIC UNION.

The regular monthly meeting of the New York Homœopathic Union was held November 19th, 1896, at 62 West Forty-ninth Street, New York, the President, Edmund Carleton, M. D., in the chair.

Members present: Doctors Finch, Gerrard, Gillingham, Littell, Munson, Richards, Vondergoltz, and Young.

The Secretary, Dr. Wilcox, sent a letter of regret, being unavoidably detained.

Dr. Young was appointed Secretary *pro tem.*

Reading from Hahnemann's *Chronic Diseases* was then taken up (pages 170–173, Hempel's edition; pages 133–136, Tafel's edition).

Dr. Finch—Hahnemann uses the word "specific" here in a specific sense; that is to say, the remedy is selected according to specific indications in the case under treatment.

Dr. Vondergoltz declaimed against the use of specifics for diseases. Each individual case must be differentiated.

Dr. Finch related the case of a boy suffering from intermittent fever, contracted while living in a damp, unhealthy cellar. The doctor cured this case without causing the boy to be removed from the cellar; and he continued to live in the cellar for years afterward in health.

Dr. Carleton—If a case of intermittent fever is really cured while the patient continues to live in the unhealthy locality, thenceforward that patient has immunity from the malady. Homœopathy only can do this, and we have many clinical cases in verification.

Dr. Vondergoltz—Recently had a patient suffering from intermittent fever complicated with massive doses of Quinine, Iron, Arsenic, and other drugs. He "took" the case carefully, and found the symptoms corresponding to Cinchona. He gave that remedy, in potency, apparently with complete success.

Dr. Carleton—Probably Cinchona was the original similar. Next spring there may be some return of the symptoms; but it will be a clear case then, and the properly selected remedy will work a perfect cure.

Dr. Gillingham considers the symptoms of the Apyraxia the most important of all, and makes his remedy tally with them. In Natrum-muriaticum cases the *time of attack* is characteristic.

Dr. Young—Had a case of twelve years, periodically returning, and always worse during each gestation, and found Lycopodium, indicated for both chronic and acute state. Eupatorium-perfoliatum was rather similar; but the vomiting between chill and fever was *sour* instead of *bitter*. Lycopodium cured the entire case. In his estimation intermittent fever requires as much skill and judgment in prescribing as any sickness to which the human system is liable.

Then followed a general discussion of Hahnemann's statement that symptoms which appear last are the first to disappear under appropriate treatment, while the old, original symptoms are last to go, and when they are gone the patient is well. The word "suppressed" in Hempel's translation was judged to mean "disappeared," but the case is *wholly* cured only by antipsorics. A number of members have often made this succession: Sulphur, Calcarea, Lycopodium.

Dr. Gillingham recalled his remarks made last spring, that he had learned from Dr. Bayard that Sulphur is chronic Rhus, and Calcarea is chronic Sulphur. He instanced one striking verification of this in a case of rheumatism, resulting in a brilliant cure.

Dr. Young worked out, according to Bœnninghausen, a case of chronic rheumatism, worse from motion. The result was always Sulphur. That remedy caused a steady diminution of the symptoms. One symptom, not in the schedule, remained unimproved by Sulphur. It is this: worse when going downstairs.

Also a case of Bright's disease, complicated with a diseased heart and tobacco poisoning. It was a perfect picture of Rhus. Sulphur being chronic Rhus, he gave it with success, thus bearing out Dr. Gillingham's statement.

Dr. Finch told of an old lady having dropsy, due to an ovarian trouble. She was a confirmed Opium eater. Her customary quid was a chunk about the size of a hickory nut. It was an Apis case. He gave the remedy, and for a time did not dare to stop the Opium. When she was somewhat improved he tried to stop the Opium, but without success, as she became about crazy, and he was obliged to allow her to resume eating Opium. Apis cured the dropsy all the same.

There was much interesting discussion of the following paragraph:

"About the period when the cure is half completed, the psoric miasm begins to become again latent; the symptoms decrease more and more, until at last mere vestiges of the psoric miasm are perceptible to the acute observer. But these, too, must be eradicated, for the least remains of a germ may eventually reproduce the disease."*

"If we were to rely upon the disease curing itself, as common people, and even the better classes of the public do, we should be very much mistaken. For little by little a new chronic disease is developed out of the remaining portion of the psoric miasm, and gradually conquers the organism." The consensus of opinion was that this stage in the treatment of a case is the most critical of all, requiring the nicest judgment on the part of the physician, when even the slightest error would result in mischief almost impossible to repair.

Dr. Vondergoltz mentioned the difficulties he encountered in the treatment of chronic gynæcological cases. When put upon suitable hygienic regimen they would become constipated. They would then become clamorous for relief from the constipation. This, as well as other symptoms, would be cured by the appropriate remedy, but he found it difficult to keep them from resorting to palliatives for constipation. It is best not to stop bad habits all at once, but do it gradually.

Dr. Carleton—Yes; Hahnemann said stop gradually.

Dr. Finch—If they stop coffee they become constipated. They take coffee again and the constipation disappears.

The meeting then went into consideration of clinical cases.

Dr. Vondergoltz presented the following report, made by his assistant, Dr. L. Beecher, 43 West Sixtieth Street:

Mrs. V., aged thirty-five years, came to my private dispensary seven months ago. One year previously she had both ovaries removed by an eminent old school surgeon. Since then she has been obliged to wear tube and rubber pouch to hold urine from right kidney. The surgeon had told her she would have to wear this arrangement always, or until he had removed the right kidney, and offered to perform this second operation, which she declined. Every month, she said, she had a gather-

* "In the same way a polypus in the water, though several of its branches may have been cut off, produces new branches in the course of time."

ing of bad-smelling pus, exuding from the small fistula in abdomen, just above the ureter and into which the rubber tube was inserted. She had a great many Sulphur symptoms, and I gave her Sulph.[30], which she took for a week, every day. She then returned to me, and said that all of her general symptoms were much better, except that the itching on her body was intense. I then gave her Sulph.[200], the highest I had.

The following week she came to the dispensary, and said that the urine had entirely ceased running from the tube into the bag, but came through the normal passage, and in sufficient quantity to show that both kidneys were acting. This astonished me greatly, as I had not undertaken to cure this seemingly surgical case.

For two weeks the urine passed from her normally, and then it went back to its former conditions—*i. e.*, one kidney (left) acting normally, the urine from the other (right) passing into the tube and bag. I was greatly mystified, and arranged an interview for her with Dr. Eric Vondergoltz at his office, where he examined her carefully, and then prescribed Sulphur again—this time in the thousandth potency, one powder to be taken immediately.

I did not see her for nearly four months afterward. Last week she came to my dispensary, and said that for three months past the urine had been passing normally from both kidneys, and the rubber tube and bag were useless. She felt herself quite cured.

Physical examination by Dr. Vondergoltz: "Woman emaciated; abdomen flat; skin yellow; in the linea alba a scar about two and one-half inches long; at the lower end of the scar a fistulous opening, all skin around bluish-red and excoriated. After having removed the inserted tube I was able to penetrate, with a Kelly sound, far up to a certain point to the right; while leaving the sound there, through the sound the urine, relatively clear, was dripping out. The patient was very sensitive; never mind; I succeeded in establishing the fact that the urine and pus were not issuing from the bladder, as the strictest examination and washing out of the bladder did not reveal the least of particles or pus, so abundantly issuing from the fistulous opening. As it is implicitly my usage to give a remedy to see the action, so in this case also, my dosage, 1,000, was regarding her nervous character. I, myself, recommended to have this done so soon as possible."

This case and its treament greatly interested all present.

Dr. Young—Have any of you cured complete fistula?

Chorus—Oh! yes.

Dr. Young and Dr. Carleton related cases of fistula cured by the homœopathic remedy, without operation.

Dr. Vondergoltz gave the history of a recto-vaginal fistula, cured with Asafœtida. He worked the case out according to Bœnninghausen, and found all the symptoms under the remedy named.

Dr. Gillingham—That remedy brings to mind a case I cured last summer. Across the street from me lived two single ladies, one of them very large. Her age was thirty-six. About half-past five one morning I was aroused from sleep by screams issuing from her apartment. Soon her servant came, running, to summon me in attendance upon Miss Laura. I found her sitting by the window, alternately screaming and gasping for breath. I sternly commanded her to stop screaming, and boxed her ears, but to no purpose. I managed to elicit the fact that she felt terribly distended with gases. What I saw was that she was constantly endeavoring to get more air. I quickly put some pellets of Carbo-veg. on her tongue. Soon she became easier, and said that she had had intense abdominal distension for a number of days; and that she had not menstruated for four months. I went home and studied the *Materia Medica*, and was struck with the similarity to Asafœtida. She was a little improved next visit, but not easy. I learned that she had passed a large quantity of flatus. The following conversation ensued:

"Did you notice any particular odor to the flatus?"

"You gave me Asafœtida."

"No."

"Yes. After you left this morning I went to the closet and passed an enormous amount of gas, and it smelled of Asafœtida."

She was put upon Asafœtida$^{200}$, in water, every two hours. The next day menses appeared, and she was soon well.

Dr. Young—Which cured—Carbo-veg. or Asafœtida?

Dr. Vondergoltz—What shall I give a school teacher, aged twenty-eight, the victim of many physicians, who has persistent neuralgia of the left trigeminus; sharp pain, accompanied with a sucking sensation in the cheek? She has dyspepsia, but eats; always tired; sleeps well; lives properly; is a slender person, and likes hot applications. She has retroflexion, and prolapsus of left ovary. She is excitable. Symptoms worse from thinking of them. Her father died of syphilitic gummata of the skull. I worked out the case a number of times, and always found Nux-vomica the remedy, and Pulsatilla next to it, though palliative.

Three remedies were suggested by different members: Calc-phos., Phos., Sepia.

Dr. Richards contributed the following history:

A maiden lady of forty-eight. Six sisters died of phthisis; father of fistula; mother of melancholia. Her life has been one of invalidism. Bad fall when three years old. Tearing pain in limbs, worse at night, cannot bear limbs against each other, gets only cat naps; slight cough. Takes cold easily. Fond of sweets. Large, blue hæmorrhoids; rectum wholly inactive; strains terribly at stool, and afterward exhausted. Yellow skin, covered with sticky sweat. Hot flashes. Urinates three or more times every night. Has always worn pessary. Examination of chest shows it to be all right. Examination of abdomen and pelvis shows retroflexion, with adhesions; ovary hardly to be felt, owing to hard ligaments; os uteri hard and contracted. Working the case out according to Bœnninghausen, Rhus-tox. proved to be the remedy, which has greatly benefited the patient. Now improvement has ceased, and the question is, whether medicine can do much more until the retroflexion is corrected.

Dr. Carleton—Probably not.

It being ten o'clock, the meeting adjourned.

CHARLES H. YOUNG, M. D.,
*Secretary, pro tem.*

